# An assessment of bioterrorism competencies among health practitioners in Australia

**DOI:** 10.3134/ehtj.09.007

**Published:** 2010-03-25

**Authors:** DV Canyon

**Affiliations:** Disaster Health and Crisis Management Unit, Anton Breinl Centre for Public Health and Tropical Medicine, School of Public Health and Tropical Medicine, James Cook University, Townsville, Australia

## Abstract

Public health and medical professionals are expected to be well prepared for emergencies, as they assume an integral role in any response. They need to be aware of planning issues, be able to identify their roles in emergency situations, and show functional competence. However, media perceptions and non-empirical publications often lack an evidence base when addressing this topic. This study attempted to assess the competencies of various health professionals by obtaining quantitative data on the state of bioterrorism preparedness and response competencies in Australia using an extensive set of competencies developed by Kristine Gebbie from the Columbia University School of Nursing Center for Health Policy with funding from the US Centres for Disease Control and Prevention. These competencies reflect the knowledge, capabilities, and skills that are necessary for best practice in public health. Sufficient data were collected to enable comparison between public health leaders, communicable disease specialists, clinicians (with and without medical degrees), and environmental health professionals. All health professionals performed well. However, the primary finding of this study was that clinicians consistently self-assessed themselves as lower in competence, and clinicians with medical degrees self-assessed themselves as the lowest in bioterrorism competence. This has important implications for health professional training, national benchmarks, standards, and competencies for the public health workforce.

## Introduction

Public health systems are designed to deal with a regular stream of crises that span a wide range. Health professionals are part of these systems and include, for instance, communicable disease specialists and emergency response personnel, who must be ready to respond to natural and made-made disasters. The public health system has an integral and critical role in responding to threats to public well being, and thus requires a well-prepared workforce. In Australia, a number of anti-terrorism bills were passed in 2002, and the USA passed the Public Health Security and Bioterrorism Response Act in the same year.^1^ This legislation contributed significantly to raising awareness of the need for well-trained health professionals. Subsequently, the Center for Health Policy at Columbia University developed an extensive list of bioterrorism preparedness competencies that were to feature prominently in training courses.^2^, ^3^ In 2003, a report by the Trust for America's Health was published, which prompted more training programs to improve nationwide capacity to respond to natural and unnatural emergency, disaster, and terror events.^4^ Similarly, from 2002 onwards, the Disaster Health and Crisis Management Group at James Cook University in Australia has developed an integrated and tiered suite of educational programs on disaster health management and bioterrorism in response to demand from over 400 students in Master of Public Health programs. Although some would argue that the spectre of bioterrorism is unlikely, Al-Qa'ida has, almost as a retort, called on its adherents to attempt biological war on US troops in Iraq late in 2006. In an advertisement to attract new recruits, Abu Hamza al-Muhajir, Al-Qa'ida's operational chief in Iraq at the time, reputedly said: ‘The field of Jihad can satisfy your scientific ambitions and the large American bases are good places to test your unconventional weapons, whether biological or dirty, as they call them’.^5^

In 2005, the US was awarded a D+(scale: A–F) for preparedness efforts;^6^ however, an appraisal of public health training programs in 2007 made the positive finding that public health now had a seat at the table in most ‘places’ and ‘incident command’ has become a well-known term.^7^ No such evaluation has been performed on Australian institutions and no information exists on bioterrorism competencies of Australian health professionals.

To counter media perceptions and publications lacking an evidence base, this study collected data on the self-assessed competencies of various health professionals to obtain quantitative data on bioterrorism preparedness and response competencies in Australia with the aim of identifying knowledge gaps and differences between health professions.

## Methods

In this study, 10 self-assessment surveys designed to ascertain the state of preparedness of the bioterrorism response community in Australia were made available from a University website. Although the use of a self-report survey is open to criticism, a well-constructed survey can provide accurate and valuable information.^8^ The sampling strategy represents a combination of criterion-based and convenience approaches.^9^ The targeted participants were the bioterrorism response community (Table 1). Questionnaire responses were collected electronically with security preserved through encryption and a secure server line.^10^

**Table 1:** Bioterrorism competency surveys and relevant health-related occupations in each survey

*Category*	*Relevant occupations*
Public health leaders	Department Head, Bureau Chief, Division Chief, Director, and Deputy Director
Clinical staff	Nurse, dentist, physician—anyone providing direct clinical care in a public health setting
Public health communicable disease staff	Specifically, outbreak investigator and epidemiologist, but includes those working with health outcomes, program evaluation, immunization, disease identification, and prevention
Environmental health staff	Specialists in research, environmental health, food, soil and plants, air pollution, hazardous materials, toxicologist, water/waste water/solid waste specialist, sanitarian, and entomologist
Public health laboratory staff	Microbiologist, chemist, toxicologist, physicist, virologist, entomologist, and non-specified laboratory professionals with a minimum qualification of a BSc
Coroner	Professionals responsible for providing legally defensible determinations of the cause of death
Public health information staff Other public health professional staff	Expert in public relations, media relations, advocacy, health promotion spokesperson Professional occupations not described above, such as health educators, legal professionals, financial officers, and others
Technical and other support staff	Bookkeepers, clerks, court workers, dispatchers, license distributors, office machine and computer operators, telephone operators, legal assistants, etc.
Public health medicine specialists	Workers in health protection, risk management, and infectious diseases

In the first nine surveys, preparedness was evaluated using 100 competencies devised by the Center for Health Policy at Columbia University with a grant from the US Centers for Disease Control and Prevention (CDC).^2^ The content of the tenth survey for public health medicine specialists comprised 15 competencies derived from an Australasian Faculty of Public Health Medicine document.^11^ These self-assessment survey tools were designed to help participants to become aware of the readiness competencies; help participants reflect on their abilities; and help guide the development of profession-oriented training materials.

The survey homepage presented information and links, which led initially to an online consent form and then to the anonymous surveys. Demographics collected included country/state of residence, occupation, qualification, and employer. On completion, survey responses were emailed automatically to the investigator. A popup screen was then generated to provide a summary of participant performance in the categories of prevention, preparedness, response, and recovery.

Recruitment took place by emailing postgraduate students, both current and alumni, who were affiliated with the Anton Breinl Centre at James Cook University from the years 2004 to 2006. The advertisement to participate was sent to 445 valid email addresses. A low response rate was expected from this pool, because how many of these were no longer active was unknown. This population was comprised primarily of mid-career doctors (50%) and other health professionals, including nurses, epidemiologists, environmental health officers, and administrators. Public health leader participation was also sought through direct communication with Chief Health Officers representing the states and territories in Australia. Thus, the results were expected to represent baseline competencies amongst health professionals who had already received public health training. Ethics approval H2328 was granted for this study by the James Cook University.

## Results

**Figure 1:**
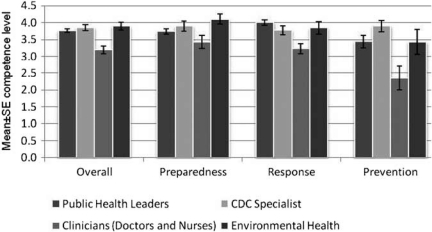
Self-assessed mean bioterrorism competency scores per participant from four different surveys on health professionals presented overall and in three primary foci (preparedness, response, and prevention). Scores were based on a Likert scale in which 1 was not competent and 5 was very competent.

A total of *77* valid responses were received with sufficient representation in four surveys to make a statistical assessment (Table 2). The four surveys were public health leader (*n*=6), communicable disease control (CDC) specialist (*n*=6), clinician (*n*=39), and environmental health practitioner (*n*=7). When overall response data were split into primary foci (preparedness, response, and prevention), several patterns became clear (Figure 1). Statistical comparison of competencies between survey types was not possible, because each survey type included a different set of questions. Public health leaders, CDC specialists, and environmental health practitioners self-assessed fairly similarly in preparedness and response categories, and in the overall category, but CDC specialists indicated having more competence in prevention. It is to be noted that clinicians under assessed their competency in all categories Table 3.

**Table 2:** Comparison of mean±s.d. scores in four surveys (Leaders, CDC, clinical and EnvHealth)

*Competency focus*	*Survey type*	*No. of assessed competencies*	*Mean*^*a*^±*s.d.*	*Top-score*
Preparedness	Leaders	25	3.62±0.58	4.7
planning				
	CDC	6	4.33±0.58	3.0
	Clinical	5	3.63±0.83	0.9
	EnvHealth	6	4.17±0.58	2.0
Preparedness roles	Leaders	10	3.92±0.59	2.3
	CDC	11	3.74±0.77	3.0
	Clinical	9	3.37±0.74	0.9
	EnvHealth	10	3.86±0.87	3.1
Preparedness	Leaders	8	3.69±0.45	1.0
communication				
	CDC	3	3.61±0.65	0.7
	Clinical	2	3.31±0.73	0.3
	EnvHealth	2	4.29±0.40	1.0
Response actions	Leaders	6	3.89±0.54	1.3
	CDC	6	3.61±0.55	2.0
	Clinical	10	3.33±0.89	1.2
	EnvHealth	3	3.71±0.78	0.6
Response roles	Leaders	1	4.17±0.00	0.2
	CDC	1	3.83±0.00	0.2
	Clinical	1	3.05±0.00	0.1
	EnvHealth	1	3.86±0.00	0.1
Response	Leaders	12	3.95±0.51	3.2
communication				
	CDC	3	3.89±0.70	1.0
	Clinical	2	3.29±0.71	0.2
	EnvHealth	3	3.95±0.74	0.7
Prevention	Leaders	4	3.71±0.42	0.7
surveillance				
	CDC	8	3.63±0.78	1.7
	Clinical	1	2.36±0.00	0.1
	EnvHealth	1	3.43±0.00	0.3
Prevention	Leaders	2	3.17±0.47	0.2
diagnostic/lab actions				
	CDC	2	4.17±0.24	0.8
	Clinical	0	0	0.0
	EnvHealth	0	0	0.0

Abbreviation: CDC, communicable disease control.

a Statistical comparison of competencies in between survey types was not possible, because each survey type included a different set of questions. Data in the primary foci (preparedness, prevention, and response) were separated into eight secondary foci (planning, roles, communication, actions, surveillance, and diagnostic/lab actions). Scores were based on a Likert scale in which 1 was ‘not competent’ and 5 was ‘very competent’. ‘Top-score’ indicates the mean number of times a competence of 5 was indicated per participant. A total of 15 out of the 90 competencies covered in the four surveys were in common to these health professions. A statistical comparison is presented in Table 3.

**Table 3:** Statistical comparison of competencies (mean (s.d.)) that were common to all professions

*Competency*	*Leader* (*n*=6)	*CDC* (*n*=*6)*	*Clinician* (*n*=39)	*EnvHealth* (*n=*7)	P*-value*
01 Identifying the agency emergency response plan	4.00 (0.63)a	4.33 (0.82)a	*3.59 (1.16)a*	*4.71 (0.49)a*	0.043
03 Demonstrating the correct use of all emergency communication equipment	3.83 (0.75)a	3.00 (1.10)a	3.21 (1.26)a	4.00 (1.16)a	0.259
04 Demonstrating my functional role(s) in emergency response drills	4.17 (0.41)ab	3.83 (0.98)ab	*3.28 (1.17*)a	*4.57 (0.54*)b	0.014
05 Implementing my individual bioterrorism response functional role	*4.17 (0.41)a*	3.83 (0.98)a	*3.05 (1.17*)a	3.86 (0.69)a	0.031
06 Maintaining regular communication with partners in other agencies involved in emergency response	3.60 (0.55)ab	3.80 (1.79)ab	*2.92 (1.11)a*	*4.29 (0.49*)b	0.015
08 Conducting workforce bioterrorism preparedness programs	3.83 (0.75)a	3.83 (1.17)a	3.46 (1.02)a	4.14 (1.07)a	0.357
16 Using established communication systems for coordination among response community during a bioterrorism event, including those for privileged information	4.00 (0.63)a	4.33 (0.52)a	3.67 (0.96)a	4.29 (0.49)a	0.140
26 Describing the public health role in emergency response in a wide range of emergencies that might arise	4.17 (0.75)a	3.83 (1.47)a	3.77 (0.81)a	3.29 (1.11)a	0.392
27 Describing your functional role(s) in emergency response	*4.33 (0.52)b*	4.00 (1.27)ab	*3.08 (0.98*)a	3.43 (0.98)ab	0.013
28 Identifying your functional role in the agency's bioterrorism response plan	4.00 (0.63)ab	*4.17(0.41)b*	*3.13 (0.92)a*	3.86 (0.90)ab	0.007
29 Describing the chain of command in emergency response	3.83 (0.75)ab	*3.67 (0.82)a*	*3.28 (1.05*)a	*4.71 (0.49*)b	0.006
30 Describing communication role(s) in emergency response within the agency using established communications systems, with the media, general public, and family, neighbors	4.20 (0.45)ab	3.67 (1.03)ab	*3.41 (0.91*)a	*4.57 (0.54*)b	0.008
31 Recognizing unusual events that might indicate an emergency and describing appropriate action	4.17 (0.41)a	4.33 (0.82)a	3.56 (1.05)a	4.14 (0.69)a	0.121
32 Applying creative problem solving and flexible thinking to unusual challenges within your functional responsibilities and evaluating effectives of all actions taken	4.17 (0.75)a	3.83 (1.60)a	3.46 (1.07)a	4.43 (0.53)a	0.103
33 Identifying limits to your own knowledge and identifying key system resources for referring matters that exceed those limits	4.17 (0.75)a	4.17 (0.98)a	*3.46 (0.94*)a	*4.29 (0.49*)a	0.037

All *P*-values refer to (overall) simple factor analysis of variance results; significant overall *P*-values (*P*<0.05) are shown in italics. *Post-hoc* Duncan tests (adjusting for multiple pair-wise comparisons by holding an overall significance level of 0.05) were carried out to assess significant differences between any two groups; if measurements were found to be significantly different between two groups (as indicated by alphabets a and b), those values are also shown in italics.

When these results were further split into eight secondary categories, additional patterns emerged (Table 2). Health leaders consistently appraised themselves as more competent than other groups in the categories of ‘preparedness roles’, ‘response actions’, ‘response roles’, and ‘prevention surveillance’, but scored lowest in ‘preparedness planning’. Examination of top scores indicated that health leaders excelled in ‘preparedness planning’ and ‘response communication’. CDC specialists appraised themselves with the highest scores in ‘preparedness planning’ and ‘prevention diagnostics/lab action’. Clinicians consistently indicated lower competence when compared with all other health professionals in every category assessed except preparedness planning. Environmental health professionals (EnvHealth) assessed themselves consistently as more competent than other groups in both communication categories. The number of top scores (Likert score 5) are noted in each category to enable comparison of the subsets of professionals who consider themselves highly competent. When top-score frequencies were graded according to their relative position and summed up, the following order was observed: CDC (28), Leaders (22), EnvHealth (18), and Clinicians (11).

**Figure 2:**
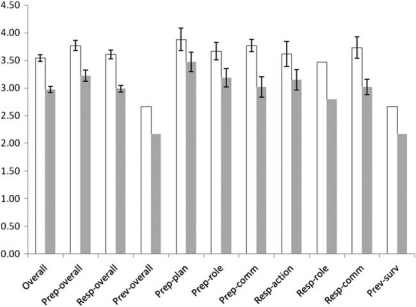
Self-assessed clinician competencies in all the pooled categories separated by those with medical degrees (shaded) and those with nursing degrees (not shaded).

There were too few responses in the Leader, CDC, and EnvHealth surveys to permit analysis of demographic variables. However, there were sufficient Clinician data to permit further analysis. Place of Clinician employment (government *n*=34, non-government *n*=5) was tested using a *t*-test and was found to be non-significant in each of the competency categories. Differences between state of residence were likewise determined by one-way analysis of variance to be non-significant. Clinicians with medical degrees (*n*=24) were compared with clinicians with nursing degrees (*n*=15) using a *t*-test. Those with medical degrees were observed to consistently assess themselves as possessing significantly less bioterrorism competencies than nurses (Figure 2).

## Discussion

To facilitate comparisons between the four survey types, competencies were pooled into the categories of preparedness, response, and prevention. All competencies in the survey were standard job competencies for any bioterrorism-related occupation. Preparedness and response were separated further into areas of activity, role awareness, and communication, whereas prevention was separated into surveillance and diagnostics.

Overall differences were minimal and surveys ranged from a score of 3.19±0.65 to 3.89±0.56 out of a 5-point Likert scale (Table 2). A very good score would have been over 4.0, but scores over 4.0 are rare.^12^ The only scores over 4.0 in the overall categories were for environmental health practitioners in preparedness and health leaders in response. In a study on general public health competencies in the USA, environmental health workers were shown to be no more competent than other health workers.^12^ However, in the USA, nurses can have ‘environmental health’ roles, hence these data were not necessarily comparable with Australian data. A search of all major publication databases resulted in no articles that surveyed ‘environmental health’ competencies in Australia. In this study, the exceptional results for environmental health professionals indicate that they should be involved in activities taking place before, during, and after bioterrorism events. It may well be beneficial to overall preparedness and response efforts if working relationships are extended by developing stronger operational and communication ties between health and environmental health responders.

Indicating oneself as a professional in a health leadership position was supported by a higher-than-average perceived level of competencies, but no exceptional competence was evident. However, Health Leaders scored very well in ‘top scores’ and the data indicates a subset of leaders who self-assessed as exceptionally competent.

Being a clinician with a medical degree, however, was indicative of lower levels of self-reported competence in bioterrorism. There are a number of possible explanations for this. Although it may reflect a lack of awareness or overconfidence in other groups, or indeed, more honesty in the medical group, it is more likely to reflect a heavier load of competing work priorities and greater demand by this group for more information and training. This result was not surprising given the results in one study, which found that only 20% of physicians or nurses had previous training in bioterrorism preparedness, but <15% felt able to respond effectively to a bioterrorism event.^13^ However, this did not affect their enthusiasm, as over 70% expressed willingness to assist the state in the event of a bioterrorist attack. Likewise, a survey of emergency and primary care physicians found that 43 and 21%, respectively, indicated being well prepared to have a role in the event of a bioterrorism attack.^14^ These low figures were confirmed in a survey on physicians in a major metropolitan area, which found that 91% self-assessed their level of bioterrorism knowledge as being ‘fair–poor’, 80% wanted more information, and 83% wanted training opportunities.^15^ Reporting bias based on profession is unlikely, as contradictory results were obtained in a similar survey in which physicians rated themselves higher than nurses.^13^ When general competencies of public health nurses in the USA were assessed, it was found that they did not feel more than minimally competent with scores averaging 2.5 out of 5.^16^ Our finding that Australian nurses scored much higher in a specialist area, such as bioterrorism, is indicative of good national standards. However, an average of 3.5 out of 5 still leaves room for improvement. This level of clinical competence was corroborated by a low frequency of ‘top scores’ in all categories.

Communicable disease control professionals were expected to do well in this survey because of their regular preparedness, response, and prevention activities, as part of outbreak management. However, they were overshadowed by health leaders and environmental health practitioners. CDC scores ranked third in both ‘preparedness role’ and ‘response role’ categories, but more ‘top scores’ were observed in ‘response actions’. In Australia, the CDC system is efficiently organized with ongoing monitoring, systematic responses along agreed lines, well-defined roles, and very good service delivery (Professor R Speare, personal communication).

Australia has not yet adopted a common set of public health, let alone bioterrorism, competencies, and there has been considerable resistance from most educational institutions. In 2009, a fairly generic set of competencies was tabled by the National Public Health Education and Research Program and tentatively accepted by participating public health institutions. Current efforts by Australian health departments to deliver disaster response training to a large proportion of medical and nursing staff is a significant move in the right direction and should raise the level of competencies. In other areas, competency improvements have been associated with comprehensive training events in key content knowledge tests and self-rated competencies.^17^ Emergency preparedness training has also been shown to increase responder confidence in duty performance by two to three times.^5^ The self-assessed results from this study suggest that Australian public health leaders have the necessary competencies to ensure the success of this effort; however, external validation of leaders and their teams may be required to confirm this.
